# Prenatal Cannabis Use and Maternal Pregnancy Outcomes

**DOI:** 10.1001/jamainternmed.2024.3270

**Published:** 2024-07-22

**Authors:** Kelly C. Young-Wolff, Sara R. Adams, Stacey E. Alexeeff, Yeyi Zhu, Edwin Chojolan, Natalie E. Slama, Monique B. Does, Lynn D. Silver, Deborah Ansley, Carley L. Castellanos, Lyndsay A. Avalos

**Affiliations:** 1 Division of Research, Kaiser Permanente Northern California, Oakland; 2 Department of Psychiatry and Behavioral Sciences, University of California, San Francisco; 3 California Department of Public Health, Richmond; 4 Public Health Institute, Oakland, California; 5 Regional Offices, Kaiser Permanente Northern California, Oakland

## Abstract

**Importance:**

Many studies have evaluated whether in utero cannabis exposure is associated with fetal and neonatal outcomes, yet little is known about whether prenatal cannabis use is associated with maternal health outcomes during pregnancy.

**Objective:**

To evaluate whether prenatal cannabis use is associated with maternal health outcomes during pregnancy.

**Design, Setting, and Participants:**

This population-based retrospective cohort study included pregnancies in Northern California from January 2011 to December 2019 that lasted 20 weeks or longer and were screened for prenatal cannabis use.

**Exposures:**

Prenatal cannabis use was defined as any self-reported use during early pregnancy or a positive toxicology test result based on universal screening at entrance to prenatal care (approximately 8-10 weeks’ gestation). Self-reported frequency of use (daily, weekly, monthly or less, never, unknown), use defined only by self-report, and use defined only by toxicology test results were examined.

**Main Outcomes and Measures:**

Electronic health record data were used to define the following outcomes: gestational hypertension, preeclampsia, eclampsia, gestational diabetes, gestational weight gain greater and less than guidelines, placenta previa, placental abruption, placenta accreta, and severe maternal morbidity. Adjusted risk ratios (aRRs) were calculated using a modified Poisson regression.

**Results:**

The sample (n = 316 722 pregnancies; 250 221 unique individuals) included 84 039 (26.5%) Asian/Pacific Islander, 20 053 (6.3%) Black, 83 145 (26.3%) Hispanic, and 118 333 (37.4%) White individuals; the mean (SD) age was 30.6 (5.4) years. Overall, 20 053 (6.3%) screened positive for prenatal cannabis use; 2.9% were positive by self-report, 5.3% by toxicology testing, and 1.8% by both. The frequency of cannabis use was 1930 (0.6%) daily, 2345 (0.7%) weekly, 4892 (1.5%) monthly or less, and 10 886 (3.4%) unknown. Prenatal cannabis use was associated with greater risk of gestational hypertension (aRR, 1.17; 95% CI, 1.13-1.21), preeclampsia (aRR, 1.08; 95% CI, 1.01-1.15), weight gain less than (aRR, 1.05; 95% CI, 1.01-1.08) and greater than (aRR, 1.09; 95% CI, 1.08-1.10) guidelines, and placental abruption (aRR, 1.19; 95% CI, 1.05-1.36). The pattern of results was similar when defining prenatal cannabis use only by self-report or only by toxicology testing, and associations between the frequency of prenatal cannabis use and outcomes varied with outcome.

**Conclusions and Relevance:**

The results of this cohort study suggest that prenatal cannabis use was associated with several adverse maternal health outcomes during pregnancy. Continued research is needed to understand whether characteristics of prenatal cannabis use (eg, dose, mode, and timing) moderate these associations.

## Key Points

**Question:**

Is prenatal cannabis use associated with maternal health outcomes during pregnancy?

**Findings:**

In this cohort study of 316 722 pregnancies, prenatal cannabis use was associated with increased risk of gestational hypertension, preeclampsia, weight gain greater and less than guidelines, and placental abruption as well as reduced risk of gestational diabetes. No association was found with eclampsia, placenta previa, placenta accreta, or severe maternal morbidity.

**Meaning:**

The results of this study suggest that the association between prenatal cannabis use and maternal health is complex and there is a need for continued research to understand how prenatal cannabis use affects the health of pregnant individuals.

## Teaser

This cohort study examines the association between prenatal cannabis use and maternal health outcomes during pregnancy.

## Introduction

Rates of prenatal cannabis use in the US have increased in recent years,^1^ corresponding with spreading legalization and rising perceptions of safety.^2,3^ Pregnant individuals report using cannabis to help with sleep, depression, stress, morning sickness, and pain during pregnancy, and many perceive cannabis to be a safer alternative to prescription medications.^2,4,5^ However, there is evidence that prenatal cannabis use is associated with moderate increases in the risk of adverse fetal and neonatal health outcomes (eg, lower birthweight, preterm birth, and neonatal intensive care unit admission),^6,7,8^ and national guidelines recommend that pregnant individuals abstain from using cannabis.^9^

Whereas many studies have examined how maternal prenatal cannabis use is associated with fetal and neonatal outcomes,^6,7,8,10,11,12^ less is known about the associations with maternal health during pregnancy. Cannabinoids, including δ^9^-tetrahydrocannabinol (THC), cross the placenta^13,14^ and may affect maternal health by binding to the cannabinoid receptors on the placenta, inhibiting migration of the epithelial layer of human placental amnion tissue, disrupting endogenous cannabinoid signaling and estrogen signaling, and affecting placental development and function.^15,16,17,18,19,20,21,22,23^ Further, maternal cannabis use is associated with increased peripheral vasoconstriction,^24^ raised maternal heart rate and blood pressure, and increased risk of adverse outcomes (eg, preeclampsia, hypertension).^25,26,27^

Existing research on the association between prenatal cannabis use or cannabis use disorder and maternal outcomes (eg, gestational diabetes [GD], hypertension, and placental abruption) has limitations that may explain mixed findings.^28,29,30,31,32,33^ Most studies are limited to self-reported cannabis use, which is known to underestimate use,^34,35^ and do not adequately account for confounders, such as noncannabis prenatal substance use. Further, previous studies may not be generalizable to current populations due to the changing modes of cannabis administration and increased potency of newer cannabis products.^36,37,38,39^

In this large, retrospective cohort study, we examined the association between prenatal cannabis use and maternal health outcomes among individuals in a large health care system with universal screening for prenatal cannabis use by self-report and urine toxicology testing, adjusting for a wide range of covariates, including maternal use of other substances.

## Methods

### Setting

Kaiser Permanente Northern California (KPNC) is an integrated health care delivery system that provides health care to 4.6 million patients, similar to the insured Northern California population.^40^ Institutional review board approval was obtained from KPNC with waiver of consent and Health Insurance Portability and Accountability Act authorization, and Strengthening the Reporting of Observational Studies in Epidemiology (STROBE) reporting guidelines were followed.

### Cohort and Measures

This population-based retrospective cohort study included singleton pregnancies that began (based on last menstrual period) during 2011 to 2019 and lasted 20 weeks or longer. Eligibility criteria included KPNC membership at any point during pregnancy and 1 or more KPNC prenatal visits. Pregnancies were required to have a response to the self-reported cannabis use question and a THC urine toxicology test (eFigure in Supplement 1). All measures were extracted from KPNC administrative and electronic health record data.

### Exposures

The primary exposure was based on universal screening at the entrance to prenatal care (approximately 8-10 weeks’ gestation) via a self-administered questionnaire and urine toxicology test to which patients consented to undergo (eMethods 1 in Supplement 1). Confirmatory testing for the presence of the cannabis metabolite, 11-nor-9-carboxy-δ^9^-THC, detectable for up to approximately 30 days after last use among those who use regularly, was performed by liquid chromatography-tandem mass spectrometry for all positive immunoassay results. Individuals were classified as having any prenatal use if they self-reported cannabis use since pregnancy or had a positive confirmed toxicology test result. Self-reported frequency of prenatal cannabis use was based on mutually exclusive categories of daily, weekly, monthly or less, and never; we created a category of unknown frequency among those with a positive toxicology test result without self-reported use.

### Maternal Outcomes

For all outcomes, *International Classification of Diseases, Ninth Revision (ICD-9) *and *ICD-10* diagnosis codes and other diagnostic criteria are provided in eMethods 2 in Supplement 1. Individual outcomes are described in the following sections.

#### Metabolic Outcomes

Among patients without chronic hypertension, we examined 3 hypertensive disorders of pregnancy: gestational hypertension, preeclampsia, and eclampsia. Gestational hypertension was defined using a combination of diagnoses, anti-hypertensive medication use, and blood pressure measurements. Preeclampsia and eclampsia were ascertained from inpatient diagnoses.

GD was ascertained from the KPNC Gestational Diabetes Registry,^41^ which uses diagnoses, medications, and laboratory tests throughout pregnancy to identify GD among those without pregestational diabetes. We examined gestational weight gain (GWG) that was either less or greater than the range recommended by the Institute of Medicine (2009; eMethods 2 in Supplement 1).^42^

#### Placental Outcomes and Severe Maternal Morbidity

Placenta previa and accreta were identified by inpatient diagnosis codes; placental abruption was identified by inpatient and outpatient diagnosis codes. Severe maternal morbidity was defined by the US Centers for Disease Control and Prevention criteria using inpatient and outpatient diagnosis codes for 21 pregnancy-related conditions.^43,44^

### Covariates

Data on maternal age at pregnancy onset, self-reported race and ethnicity (Asian/Pacific Islander, Black, Hispanic, White, and other, including American Indian, Alaska Native, and multiracial, which was included as a social construct due to known differences in the prevalence of prenatal cannabis use by race and ethnicity), parity (0, 1, ≥2), maternal insurance (Medicaid vs other), neighborhood deprivation index (quartiles),^45^ birth year, and prepregnancy body mass index (calculated as weight in kilograms divided by height in meters squared) were extracted from the electronic health record. Month of prenatal care initiation was classified using the Kotelchuck initiation index: inadequate (≥7), intermediate (5-6), adequate (3-4), or adequate plus (1-2).^46^ Noncannabis prenatal substance use was based on universal screening at entrance to prenatal care and defined as prenatal use of alcohol, nicotine, opioids, stimulants, and anxiety/sleep medications (eMethods 1 in Supplement 1).

For maternal comorbidities, *ICD* diagnosis codes were used to define pregestational diabetes (type 1 or 2) during the 2 years before pregnancy, mood/anxiety disorder, other psychiatric disorders, and noncannabis substance use disorder diagnoses during the year before pregnancy through the first prenatal visit and nausea and vomiting of pregnancy through the first prenatal visit. Antidepressant medication use was defined as a prescription fill during pregnancy through the first prenatal visit or before pregnancy with the supply lasting past the pregnancy onset date.

### Statistical Analysis

We fit extended modified Poisson models with robust standard errors using the extension of the sandwich variance estimator to account for the correlation in the outcomes for multiple pregnancies per person.^47^ Risk ratios (RRs) and adjusted RRs (aRRs) with 95% CIs were reported. Model 1 was unadjusted. Model 2 was adjusted for maternal sociodemographic characteristics, parity, birth year, prenatal care initiation, prepregnancy body mass index, other noncannabis prenatal substance use (ie, alcohol, nicotine, opioids, stimulants, and anxiety/sleep medications), and comorbidities (ie, pregestational diabetes, nausea/vomiting during pregnancy, mood/anxiety disorders, other psychiatric disorders, substance use disorders, and antidepressant use). We also tested for associations between use frequency and outcomes, followed by a trend test when results suggested an ordering of associations across frequency levels; trend tests excluded the category of frequency unknown, positive toxicology test result.

### Sensitivity Analyses

To assess differences in exposure classification, we fit separate models in which the exposure (ie, any prenatal cannabis use) was determined only by self-report status or urine toxicology test results. We also repeated analyses (1) that were limited to the subset of pregnancies without evidence of noncannabis prenatal substance use, (2) among those who entered prenatal care during the first trimester, and (3) after removing calendar year as a covariate, as controlling for calendar time may constitute overadjustment that could bias the estimated associations.

The standardized difference was calculated as the difference in proportions divided by the standard error.^48^ Analyses were conducted using SAS, version 9.4 (SAS Institute), and R, version 4.0.2 (R Foundation), from October 2023 to May 2024. Two-sided *P* values of <.05 were considered statistically significant.

## Results

The maternal characteristics of the 316 722 pregnancies (from 250 221 unique individuals) were 84 039 (26.5%) Asian/Pacific Islander, 20 053 (6.3%) Black, 83 145 (26.3%) Hispanic, and 118 333 (37.4%) White individuals; 49 015 (15.5%) were younger than 25 years, and 28 803 (9.1%) were insured by Medicaid. Overall, 20 053 (6.3%) screened positive for prenatal cannabis use by either self-report or toxicology testing; 2.9% were positive by self-report, 5.3% were positive by toxicology, and 1.8% were positive by both. Self-report and toxicology testing were completed at a median of 8.3 weeks (IQR,7.0-10.3) and 9.0 weeks (IQR, 7.7-11.4) of gestation, respectively. Pregnancies excluded due to missing data on prenatal cannabis use had sociodemographic characteristics like the final sample but had more missing data (eTable 1 in Supplement 1). The frequency of cannabis use was 1930 (0.6%) daily, 2345 (0.7%) weekly, and 4892 (1.5%) monthly or less, and 10 886 (3.4%) had a positive toxicology test result but did not report cannabis use (Table 1). Pregnancy characteristics by frequency are shown in eTable 2 in Supplement 1. Several maternal outcomes were rare (<5%): preeclampsia (44 363 [4.8%]), eclampsia (384 [0.1%]), placenta previa (3499 [1.1%]), placental abruption (4002 [1.3%]), placenta accreta (499 [0.2%]), and severe maternal morbidity (10 338 [3.3%]). Gestational hypertension (44 363 [14.7%]), GD (36 374 [11.7%]), and GWG less than (48 117 [16.0%]) and greater than (176 898 [58.8%]) guidelines were more common (Table 2).

**Table 1.** Pregnancy Characteristics Overall and by Prenatal Cannabis Use

Pregnancy characteristics	No. (%)			Standardized difference^a^
	Overall (N = 316 722)	Prenatal cannabis use		
		Yes (n = 20 053 [6.3%])	No (n = 296 669 [93.7%])	
Age at pregnancy onset, y				
<18	2994 (0.9)	617 (3.1)	2377 (0.8)	0.68
18-24	46 021 (14.5)	7541 (37.6)	38 480 (13.0)	
25-30	87 882 (27.7)	5469 (27.3)	82 413 (27.8)	
31-35	113 020 (35.7)	4250 (21.2)	108 770 (36.7)	
≥36	66 805 (21.1)	2176 (10.9)	64 629 (21.8)	
Self-reported race and ethnicity				
Hispanic	83 145 (26.3)	5583 (27.8)	77 562 (26.1)	0.80
Non-Hispanic				
American Indian/Alaska Native^b^	1078 (0.3)	144 (0.1)	934 (0.3)	
Asian/Pacific Islander	84 039 (26.5)	1191 (5.9)	82 848 (27.9)	
Black	20 053 (6.3)	4480 (22.3)	15 573 (5.2)	
White	118 333 (37.4)	7750 (38.6)	110 583 (37.3)	
Multiracial^b^	4540 (1.3)	622 (0.2)	3918 (1.2)	
Unknown^b^	5534 (1.8)	283 (0.1)	5251 (1.7)	
Parity				
0	129 585 (40.9)	9906 (49.4)	119 679 (40.3)	0.25
1	110 434 (34.9)	5471 (27.3)	104 963 (35.4)	
≥2	65 908 (20.8)	3408 (17.0)	62 500 (21.1)	
Unknown	10 795 (3.4)	1268 (6.3)	9527 (3.2)	
Prenatal care initiation				
Adequate plus (months 1-2)	198 083 (62.5)	11 932 (59.5)	186 151 (62.7)	0.13
Adequate (months 3-5)	99 481 (31.4)	6619 (33.0)	92 862 (31.3)	
Intermediate (months 5-6)	11 297 (3.6)	962 (4.8)	10 335 (3.5)	
Inadequate (months 7+)	7861 (2.5)	540 (2.7)	7321 (2.5)	
Insured by Medicaid	28 803 (9.1)	5049 (25.2)	23 754 (8.0)	0.47
Neighborhood deprivation index, quartile				
1 (Least deprivation)	74 902 (23.6)	2641 (13.2)	72 261 (24.4)	0.41
2	74 934 (23.7)	3987 (19.9)	70 947 (23.9)	
3	74 907 (23.7)	5315 (26.5)	69 592 (23.5)	
4 (Most deprivation)	74 916 (23.7)	7510 (37.5)	67 406 (22.7)	
Unknown	17 063 (5.4)	600 (3.0)	16 463 (5.5)	
Noncannabis substance use during pregnancy				
Alcohol	29 565 (9.3)	3987 (19.9)	25 578 (8.6)	0.33
Nicotine	14 281 (4.5)	4499 (22.4)	9782 (3.3)	0.60
Opioids	22 054 (7.0)	2561 (12.8)	19 493 (6.6)	0.21
Stimulants	1712 (0.5)	598 (3.0)	1114 (0.4)	0.20
Anxiety or sleep medications	9163 (2.9)	1370 (6.8)	7793 (2.6)	0.20
Prepregnancy BMI categories				
Underweight (<18.5)	7164 (2.3)	537 (2.7)	6627 (2.2)	0.24
Normal (18.5-24.9)	137 375 (43.4)	6945 (34.6)	130 430 (44.0)	
Overweight (25-29.9)	83 426 (26.3)	5275 (26.3)	78 151 (26.3)	
Obesity (≥30)	73 367 (23.2)	6433 (32.1)	66 934 (22.6)	
Unknown	15 390 (4.9)	863 (4.3)	14 527 (4.9)	
Diagnoses and medications				
2 y Before pregnancy				
Diabetes	4026 (1.3)	222 (1.1)	3804 (1.3)	−0.02
1 y Before pregnancy through first prenatal visit				
Mood/anxiety disorder	34 984 (11.0)	4514 (22.5)	30 470 (10.3)	0.34
Other psychiatric disorder	7498 (2.4)	1269 (6.3)	6229 (2.1)	0.21
Substance use disorder diagnosis (other than cannabis)	11 220 (3.5)	3251 (16.2)	7969 (2.7)	0.48
Pregnancy onset through first prenatal visit				
Nausea and vomiting	35 639 (11.3)	4642 (23.1)	30 997 (10.4)	0.34
Antidepressant medication use	6441 (2.0)	865 (4.3)	5576 (1.9)	0.14
Frequency of cannabis use				
Daily	1930 (0.6)	1930 (9.6)	0	1.31
Weekly	2345 (0.7)	2345 (11.7)	0	
Monthly or less	4892 (1.5)	4892 (24.4)	0	
Never	296 669 (93.7)	0	296 669 (100)	
Unknown frequency, positive toxicology results	10 886 (3.4)	10 886 (54.3)	0	
Fetal outcome				
Live birth	314 480 (99.3)	19 880 (99.1)	294 600 (99.3)	0.20
Stillbirth	1510 (0.5)	114 (0.6)	1396 (0.5)	
Therapeutic abortion	732 (0.2)	59 (0.3)	673 (0.2)	

Abbreviation: BMI, body mass index (calculated as weight in kilograms divided by height in meters squared).

^a^ Standardized difference is the difference in proportions divided by the standard error; imbalance defined as absolute value greater than 0.20 (small effect size).

^b^ These categories were collapsed in the models.

**Table 2.** Frequency and Risk Ratios of Maternal Outcomes by Any Prenatal Cannabis Use

Maternal outcomes	Pregnancies, No. (%)			Risk ratios of maternal outcomes for any prenatal cannabis use vs none (95% CI)^a^	
	Overall (N = 316 722)	Prenatal cannabis use		Model 1	Model 2
		Yes (n = 20 053)	No (n = 296 669)		
Metabolic outcomes					
Hypertensive disorders^b^					
Gestational hypertension	44 363 (14.7)	3822 (20.3)	40 541 (14.3)	1.42 (1.38-1.46)	1.17 (1.13-1.21)
Preeclampsia	14 378 (4.8)	1212 (6.4)	13 166 (4.6)	1.39 (1.31-1.47)	1.08 (1.01-1.15)
Eclampsia	384 (0.1)	34 (0.2)	350 (0.1)	1.46 (1.03-2.08)	1.17 (0.80-1.71)
Gestational diabetes^c^	36 374 (11.7)	1521 (7.7)	34 853 (11.9)	0.64 (0.61-0.68)	0.89 (0.85-0.94)
Gestational weight gain^d^					
Within guidelines	75 844 (25.2)	3284 (17.1)	72 560 (25.8)	NA	NA
Less than guidelines	48 117 (16.0)	2727 (14.2)	45 390 (16.1)	1.18 (1.15-1.21)	1.05 (1.01-1.08)
Greater than guidelines	176 898 (58.8)	13 145 (68.6)	176 898 (58.8)	1.15 (1.15-1.16)	1.09 (1.08-1.10)
Placental outcomes					
Placenta previa	3499 (1.1)	169 (0.8)	3330 (1.1)	0.75 (0.64-0.88)	1.02 (0.87-1.20)
Placental abruption	4002 (1.3)	281 (1.4)	3721 (1.3)	1.12 (0.99-1.26)	1.19 (1.05-1.36)
Placenta accreta^e^	499 (0.2)	37 (0.2)	462 (0.2)	1.20 (0.86-1.67)	1.34 (0.92-1.95)
Severe maternal morbidity	10 338 (3.3)	718 (3.6)	9620 (3.2)	1.10 (1.02-1.19)	0.97 (0.89-1.05)

Abbreviation: NA, not applicable.

^a^ Model 1: modified Poisson model with robust standard errors (no covariates). Model 2: adjusted for maternal sociodemographic characteristics (age category, race and ethnicity, neighborhood deprivation index), parity, birth year, prenatal care initiation, prepregnancy body mass index category, noncannabis prenatal substance use (alcohol, nicotine, opioids, stimulants, and anxiety/sleep medications), and maternal medical and mental health comorbidities (pregestational diabetes, nausea/vomiting during pregnancy, mood/anxiety disorders, other psychiatric disorders, substance use disorders [other than cannabis], and antidepressant use).

^b^ Pregnancies of individuals with chronic hypertension were excluded (n = 14 187).

^c^ Pregnancies of individuals with pregestational diabetes were excluded. In addition, gestational diabetes could not be ascertained for pregnancies ending in therapeutic abortion. A total of 4737 pregnancies were excluded. In model 2, pregestational diabetes was not included as a covariate for the gestational diabetes outcome.

^d^ Categories determined by the 2009 Institute of Medicine guidelines. Pregnancies with missing weight values were excluded (n = 15 863). Less than guidelines models were fit among pregnancies less than or within guidelines; greater than guidelines models were fit among pregnancies greater than or within guidelines.

^e^ Placenta accreta could only be ascertained for pregnancies that were delivered in a Kaiser Permanente Northern California facility and ended in live birth or stillbirth (alive at admission); 10 979 were excluded.

### Primary Exposure: Any Prenatal Cannabis Use

Table 2 shows the unadjusted RRs and fully aRRs for each outcome, and eTable 3 in Supplement 1 shows how results changed by including stepwise covariates. In fully adjusted models, prenatal cannabis use was associated with an increased risk of gestational hypertension (aRR, 1.17; 95% CI, 1.13-1.21) and preeclampsia (aRR, 1.08; 95% CI, 1.01-1.15) but not eclampsia (aRR, 1.17; 95% CI, 0.80-1.71) (Figure).

**Figure.**
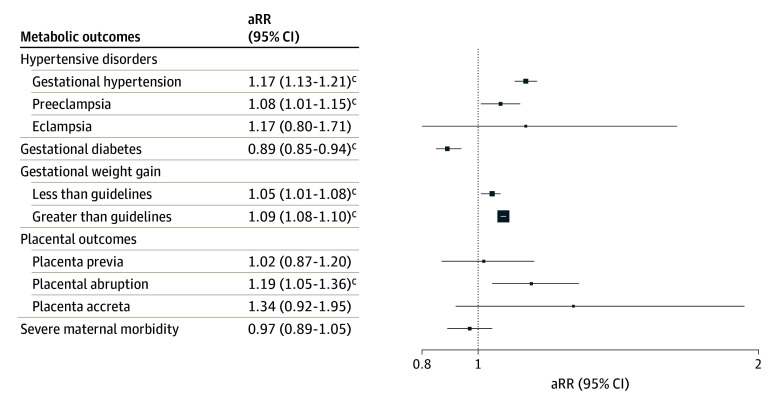
Adjusted Risk Ratios (aRRs)^a^ of Maternal Outcomes in Pregnancies With Any Prenatal Cannabis Use vs None^b^ ^a^Risk ratios were adjusted for maternal sociodemographic characteristics (age category, race and ethnicity, and neighborhood deprivation index), parity, birth year, prenatal care initiation, prepregnancy body mass index category, noncannabis prenatal substance use (alcohol, nicotine, opioids, stimulants, and anxiety/sleep medications), and maternal medical and mental health comorbidities (pregestational diabetes, nausea/vomiting during pregnancy, mood/anxiety disorders, other psychiatric disorders, substance use disorders [other than cannabis], and antidepressant use). Pregestational diabetes was not included as a covariate in the gestational diabetes model as patients with pregestational diabetes were ineligible for this outcome. ^b^Pregnancies of individuals with chronic hypertension were excluded (n = 14 187) from models of hypertensive outcomes. A total of 4737 pregnancies were excluded from the gestational diabetes model (pregnancies of individuals with pregestational diabetes were ineligible, and gestational diabetes could not be ascertained for pregnancies ending in therapeutic abortion). Pregnancies with missing weight values were excluded (n = 15 863) from the gestational weight gain models. Placenta accreta could only be ascertained for pregnancies that were delivered in a Kaiser Permanente Northern California facility and ended in live birth or stillbirth (alive at admission); 10 979 were excluded. ^c^Statistical significance at the *P* < .05 level.

In the fully adjusted models, prenatal cannabis use was associated with a significantly decreased risk of GD (aRR, 0.89; 95% CI, 0.85-0.94), an increased risk of GWG less than guidelines (aRR, 1.05; 95% CI, 1.01-1.08) and greater than guidelines (aRR, 1.09; 95% CI, 1.08-1.10), and an increased risk of placental abruption (aRR, 1.19; 95% CI, 1.05-1.36). Prenatal cannabis use was not significantly associated with placenta previa, placenta accreta, or severe maternal morbidity in fully adjusted models.

### Secondary Exposure: Frequency of Prenatal Cannabis Use

We found a dose-response association between use frequency and risk of gestational hypertension, with risk decreasing from daily (aRR, 1.24; 95% CI, 1.14-1.36) to weekly (aRR, 1.21; 95% CI, 1.11-1.31) to monthly use (aRR, 1.03; 95% CI, 0.97-1.10) vs never use (trend-test *P* < .001; Table 3). Unknown frequency (positive toxicology test result but no self-reported use) was also associated with a greater risk of gestational hypertension (aRR, 1.21; 95% CI, 1.16-1.25). No other trends were evident; however, some additional findings for frequency were statistically significant. A greater risk of preeclampsia was found for unknown frequency of use (aRR, 1.14; 95% CI, 1.06-1.24). Increased risk of GWG less than guidelines was found for weekly use (aRR, 1.09; 95% CI, 1.01-1.18), while increased risk of GWG greater than guidelines was found for daily (aRR, 1.06; 95% CI, 1.03-1.09), weekly (aRR, 1.07; 95% CI, 1.04-1.09), monthly or less (aRR, 1.06; 95% CI, 1.04-1.08), and unknown frequency (aRR, 1.11; 95% CI, 1.10-1.12). Greater risk of placental abruption was found for monthly or less use (aRR, 1.31; 95% CI, 1.03-1.67). In contrast, lower risk of GD was found for monthly or less (aRR, 0.88; 95% CI, 0.79-0.98) and unknown frequency of use (aRR, 0.89; 95% CI, 0.83-0.95). There was no association between use frequency and other outcomes.

**Table 3.** Frequency and Adjusted Risk Ratios (ARRs) of Maternal Outcomes by Frequency of Prenatal Cannabis Use

Maternal outcomes by frequency of prenatal cannabis use	Pregnancies, No. (%)	aRR (95% CI)^a^
**Metabolic outcomes**		
Hypertensive disorders^b^		
Gestational hypertension		
Daily	402 (22.3)	1.24 (1.14-1.36)
Weekly	475 (21.3)	1.21 (1.11-1.31)
Monthly or less	838 (18.0)	1.03 (0.97-1.10)
Never	40 541 (14.3)	1 [Reference]
Unknown frequency, positive toxicology result	2107 (20.7)	1.21 (1.16-1.25)
Preeclampsia		
Daily	129 (7.2)	1.15 (0.96-1.36)
Weekly	147 (6.6)	1.11 (0.95-1.30)
Monthly or less	253 (5.4)	0.89 (0.79-1.01)
Never	13 166 (4.6)	1 [Reference]
Unknown frequency, positive toxicology result	683 (6.7)	1.14 (1.06-1.24)
Eclampsia		
Daily	3 (0.2)	1.11 (0.35-3.49)
Weekly	5 (0.2)	1.53 (0.61-3.85)
Monthly or less	8 (0.2)	1.13 (0.54-2.35)
Never	350 (0.1)	1 [Reference]
Unknown frequency, positive toxicology result	18 (0.2)	1.13 (0.69-1.83)
Gestational diabetes^c^		
Daily	149 (7.8)	0.99 (0.85-1.15)
Weekly	160 (6.9)	0.87 (0.75-1.00)
Monthly or less	341 (7.1)	0.88 (0.79-0.98)
Never	34 853 (11.9)	1 [Reference]
Unknown frequency, positive toxicology result	871 (8.1)	0.89 (0.83-0.95)
Gestational weight gain^d^		
Less than guidelines		
Daily	281 (46.0)	1.05 (0.96-1.14)
Weekly	340 (46.6)	1.09 (1.01-1.18)
Monthly or less	658 (42.7)	1.04 (0.98-1.10)
Never	45 390 (38.5)	1 [Reference]
Unknown frequency, positive toxicology results	1448 (46.3)	1.04 (1.00-1.08)
Greater than guidelines		
Daily	1230 (78.8)	1.06 (1.03-1.09)
Weekly	1481 (79.2)	1.07 (1.04-1.09)
Monthly or less	3070 (77.6)	1.06 (1.04-1.08)
Never	163 753 (69.3)	1 [Reference]
Unknown frequency, positive toxicology result	7364 (81.4)	1.11 (1.10-1.12)
**Placental outcomes**		
Placenta previa		
Daily	17 (0.9)	1.10 (0.68-1.77)
Weekly	26 (1.1)	1.38 (0.93-2.04)
Monthly or less	43 (0.9)	1.06 (0.78-1.44)
Never	3330 (1.1)	1 [Reference]
Unknown frequency, positive toxicology result	83 (0.8)	0.93 (0.74-1.16)
Placental abruption		
Daily	29 (1.5)	1.26 (0.87-1.84)
Weekly	29 (1.2)	1.07 (0.74-1.55)
Monthly or less	74 (1.5)	1.31 (1.03-1.67)
Never	3721 (1.3)	1 [Reference]
Unknown frequency, positive toxicology result	149 (1.4)	1.15 (0.98-1.37)
Placenta accreta^e^		
Daily	4 (0.2)	1.51 (0.54-4.22)
Weekly	5 (0.2)	1.60 (0.64-4.04)
Monthly or less	7 (0.1)	1.10 (0.51-2.38)
Never	462 (0.2)	1 [Reference]
Unknown frequency, positive toxicology result	21 (0.2)	1.36 (0.85-2.16)
**Severe maternal morbidity**		
Daily	76 (3.9)	1.00 (0.80-1.26)
Weekly	77 (3.3)	0.88 (0.70-1.10)
Monthly or less	180 (3.7)	1.00 (0.86-1.16)
Never	9620 (3.2)	1 [Reference]
Unknown frequency, positive toxicology result	385 (3.5)	0.97 (0.87-1.07)

^a^ Modified Poisson models with robust standard errors were adjusted for maternal sociodemographic characteristics (age category, race and ethnicity, neighborhood deprivation index), parity, birth year, prenatal care initiation, prepregnancy body mass index category, other noncannabis prenatal substance use (alcohol, nicotine, opioids, stimulants, and anxiety/sleep medications), and maternal medical and mental health comorbidities (pregestational diabetes, nausea/vomiting during pregnancy, mood/anxiety disorders, other psychiatric disorders, substance use disorders [other than cannabis], and antidepressant use).

^b^ Pregnancies of individuals with chronic hypertension were excluded (n = 14 187).

^c^ Pregnancies of individuals with pregestational diabetes were excluded. In addition, gestational diabetes could not be ascertained for pregnancies ending in therapeutic abortion. A total of 4737 pregnancies were excluded. Pregestational diabetes was not included as a covariate.

^d^ Categories determined by the 2009 Institute of Medicine guidelines. Pregnancies with missing weight values were excluded (n = 15 863). Less than guidelines models were fit among pregnancies less than or within guidelines; greater than guidelines models were fit among pregnancies greater than or within guidelines.

^e^ Placenta accreta could only be ascertained for pregnancies that were delivered in a Kaiser Permanente Northern California facility and ended in live birth or stillbirth (alive at admission); 10 979 were excluded.

### Sensitivity Analyses

Results followed the same general pattern when defining prenatal cannabis use by self-report or toxicology testing only (Table 4). Associations for most outcomes were slightly stronger for toxicology tests than for self-report. Results from sensitivity analyses limited to pregnant individuals without evidence of noncannabis prenatal substance use followed the same general pattern but were slightly stronger than the main models (eTable 4 in Supplement 1). Results from sensitivity analyses that were limited to individuals who entered prenatal care during the first trimester and analyses that did not include calendar year as a covariate were similar to the main results (eTable 4 in Supplement 1).

**Table 4.** Frequency and Adjusted Risk Ratios (aRRs) of Maternal Outcomes by Prenatal Cannabis Use Defined Only by Self-Report or Defined Only by Toxicology Testing

Outcome	Overall (N = 316 722 [100%])	Prenatal cannabis use defined only by self-report			Prenatal cannabis use defined only by toxicology testing		
		Any prenatal cannabis use, No. (%)		aRR (95% CI)^a^	Any prenatal cannabis use, No. (%)		aRR (95% CI)^a^
		Yes (n = 9167 [2.9%])	No (n = 307 555 [97.1%])		Yes (n = 16 638 [5.3%])	No (n = 300 084 [94.7%])	
Metabolic outcomes							
Hypertensive disorders^b^							
Gestational hypertension	44 363 (14.7)	1715 (19.8)	42 648 (14.5)	1.10 (1.05-1.15)	3511 (22.5)	41 614 (14.5)	1.19 (1.15-1.23)
Preeclampsia	14 378 (4.8)	529 (6.1)	13 849 (4.7)	0.99 (0.90-1.08)	1016 (6.5)	13 362 (4.7)	1.11 (1.04-1.19)
Eclampsia	384 (0.1)	16 (0.2)	368 (0.1)	1.21 (0.70-2.09)	27 (0.2)	357 (0.1)	1.09 (0.72-1.65)
Gestational diabetes^c^	36 374 (11.7)	650 (7.2)	35 724 (11.8)	0.91 (0.84-0.98)	1279 (7.8)	35 095 (11.9)	0.89 (0.84-0.94)
Gestational weight gain^d^							
Within guidelines	75 844 (25.2)	1603 (18.5)	74 241 (25.4)	NA	2636 (16.5)	73 208 (25.7)	NA
Less than guidelines	48 117 (16.0)	1279 (14.8)	46 838 (16.0)	1.05 (1.00-1.09)	2296 (14.4)	45 821 (16.1)	1.05 (1.01-1.08)
Greater than guidelines	176 898 (58.8)	5781 (66.7)	171 117 (58.6)	1.05 (1.04-1.06)	11 021 (69.1)	165 877 (58.2)	1.10 (1.09-1.11)
Placental outcomes							
Placenta previa	3499 (1.1)	86 (0.9)	3413 (1.1)	1.15 (0.92-1.44)	136 (0.8)	3363 (1.1)	1.01 (0.84-1.20)
Placental abruption	4002 (1.3)	132 (1.4)	3870 (1.3)	1.22 (1.01-1.47)	236 (1.4)	3766 (1.3)	1.19 (1.03-1.37)
Placenta accreta^e^	499 (0.2)	16 (0.2)	483 (0.2)	1.28 (0.74-2.20)	34 (0.2)	465 (0.2)	1.44 (0.98-2.13)
Severe maternal morbidity	10 338 (3.3)	333 (3.6)	10 005 (3.3)	0.97 (0.87-1.09)	593 (3.6)	9745 (3.2)	0.96 (0.88-1.05)

Abbreviation: NA, not applicable.

^a^ Reference is no prenatal cannabis use. Modified Poisson models with robust standard errors were adjusted for maternal sociodemographic characteristics (age category, race and ethnicity, neighborhood deprivation index), parity, birth year, prenatal care initiation, prepregnancy body mass index category, other noncannabis prenatal substance use (alcohol, nicotine, opioids, stimulants, and anxiety/sleep medications), and maternal medical and mental health comorbidities (pregestational diabetes, nausea/vomiting during pregnancy, mood/anxiety disorders, other psychiatric disorders, substance use disorders [other than cannabis], and antidepressant use).

^b^ Pregnancies of individuals with chronic hypertension were excluded (n = 14 187).

^c^ Pregnancies of individuals with pregestational diabetes were excluded. In addition, gestational diabetes could not be ascertained for pregnancies ending in therapeutic abortion. A total of 4737 pregnancies were excluded. Pregestational diabetes was not included as a covariate.

^d^ Categories determined by the 2009 Institute of Medicine guidelines. Pregnancies with missing weight values were excluded (n = 15 863).

^e^ Placenta accreta could only be ascertained for pregnancies that were delivered in a Kaiser Permanente Northern California facility and ended in live birth or stillbirth (alive at admission); 10 979 were excluded.

## Discussion

The relative lack of research on how prenatal cannabis use is associated with maternal health vs offspring health is notable. This study leveraged data from a large health care system to examine the associations between cannabis use during early pregnancy and maternal health, adjusting for clinical and sociodemographic factors. Prenatal cannabis use was associated with greater risk of gestational hypertension, preeclampsia, GWG outside of guidelines, and placental abruption and lower risk of GD. Prenatal cannabis use was associated with a greater risk of eclampsia, but the result did not reach statistical significance, possibly due to the rarity of this outcome. Associations were slightly stronger for most outcomes when examining cannabis use measured by toxicology testing vs self-report, but the pattern was similar across exposure definitions. Further, results followed a similar pattern when the sample was limited to individuals without any noncannabis substance use during pregnancy, suggesting that results were not due to co-occurring substance use. The disparities in prenatal cannabis use by race and ethnicity, age, and neighborhood deprivation have the potential to exacerbate existing inequities in maternal health outcomes during pregnancy.

Our finding of greater risk of gestational hypertension and preeclampsia associated with prenatal cannabis use differs from prior studies that found no association or an inverse association between prenatal cannabis use based on self-report or urine toxicology testing and these outcomes.^30,31,33,49,50^ However, the results were consistent with several studies that defined prenatal cannabis exposure based on cannabis-related diagnoses,^28,51^ reflecting heavier or problematic use. For example, a large, retrospective cohort study of California hospital discharge data found that pregnant individuals with vs without a cannabis use disorder diagnosis had higher risk of gestational hypertension (aRR, 1.19; 95% CI, 1.06-1.34) and preeclampsia (aRR, 1.16; 95% CI, 1.04-1.28).^51^ In our study, there was evidence of a dose-response association between the frequency of self-reported cannabis use and gestational hypertension, with the greatest risk associated with daily cannabis use. Together, the findings suggest that more frequent prenatal cannabis use may motivate these associations, and differences in exposure measurement may partially explain the inconsistencies in the limited existing literature. Studies of US adults have found that cannabis use, especially more frequent use, was associated with increased odds of myocardial infarction and stroke.^52,53,54,55^ Future studies are needed to determine if associations with gestational hypertension and preeclampsia are replicable. If so, research could evaluate whether individuals with prenatal cannabis use might benefit from interventions for preventing preeclampsia (eg, low-dose aspirin).

Prenatal cannabis use was also associated with greater risk of GWG that is greater than guidelines, which is associated with hypertensive disorders of pregnancy,^56^ and could partially mediate the association between prenatal cannabis use and gestational hypertension and preeclampsia. However, prenatal cannabis use was also associated with GWG that was less than guidelines, even after adjusting for potentially confounding factors (eg, nausea and vomiting). GWG outside of recommendations is associated with health issues for pregnant individuals and their children,^42^ and future studies are needed to explore its potential mediating effects.

Prior studies have found inconsistent associations between prenatal cannabis use and GD. We found a lower risk of GD associated with prenatal cannabis use that was consistent with 3 retrospective cohort studies that found an inverse association between prenatal cannabis use or cannabis-related diagnoses and GD.^30,32,33^ Other studies have found no association or a positive association between prenatal cannabis use and GD.^31,57^ Results were consistent with a recent meta-analysis in adults that found a lower risk of developing type 2 diabetes among individuals with vs without cannabis use (aRR, 0.48; 95% CI, 0.39-0.59).^58^ Hypothesized mechanisms included cannabis-related attenuated inflammatory response, stress signaling, and reactive oxygen species formation, which may be stronger for nonsmoked modes of administration given the potential of smoking to increase oxidative stress.^58^ In frequency analyses, a statistically significant lower risk was only found among those who self-reported using monthly or less, which does not provide evidence for a dose-response association. Future research is also needed to understand the potential mechanisms underlying lower risk of GD among those who use cannabis infrequently during early pregnancy, who may be different in key unmeasured ways that we were unable to adjust for, and test whether findings differ based on modes of use.

Whereas several prior studies have not found an association between prenatal cannabis use and placental abruption,^31,33,49^ our finding of a greater risk of placental abruption being associated with prenatal cannabis use was consistent with results from a prior large, population-based pregnancy cohort study of self-reported cannabis use with a matched design that found higher risk of placental abruption among those with vs without prenatal cannabis use (aRR, 1.72; 95% CI, 1.54-1.92).^30^ Cannabis-related diagnoses during pregnancy have been positively associated with placenta previa^32^ and severe maternal morbidity^51^; however, we did not find an association between use and placenta previa, accreta, or severe maternal morbidity.

In this and our prior studies, we have documented low sensitivity of self-reported prenatal cannabis use.^35^ There are real and perceived risks of disclosing prenatal substance use. Prenatal cannabis use can have greater consequences for individuals from disadvantaged backgrounds due to inequities in who gets tested and reported to child protective services and law enforcement,^59,60,61^ and some individuals may avoid prenatal care due to these concerns.^62,63^ In California, prenatal cannabis use is not sufficient to make a child abuse or neglect report.^64^ This allows KPNC to offer universal prenatal substance use screening and linkage to further assessment, education, and patient-centered, supportive, nonstigmatizing prenatal substance use intervention linked with prenatal care.^65,66,67^ Routine testing for prenatal cannabis use is not recommended in states with punitive policies that criminalize or penalize prenatal substance use.

### Limitations

This study had limitations. Our sample was limited to insured pregnant patients in a large health care organization in Northern California. The findings may not generalize to uninsured patients or those outside of California, a state that legalized medicinal cannabis in 1996 and adult use in November 2016 (with legal adult use sales beginning in January 2018). The prenatal cannabis use screening occurred at entrance to prenatal care, and we were unable to determine whether prenatal use occurred only before pregnancy recognition or continued later into pregnancy. It is possible that urine toxicology tests detected prepregnancy cannabis use. However, this is unlikely, given that toxicology tests were given at a median of 9.0 weeks’ gestation. Further, important aspects of cannabis use were not assessed, including the mode of administration, potency, products used, and reasons for use. Urine toxicology tests are more likely to detect heavy vs infrequent cannabis use, and the duration that cannabis is detectable could vary with mode of use. Although we adjusted for many covariates, unmeasured confounders may have affected results. Additional research is needed to determine whether the associations found in this study are causal and whether they vary depending on the trimester of use, modes of administration, and product strength.

## Conclusions

In this cohort study, prenatal cannabis use was associated with a greater risk of gestational hypertension, preeclampsia, GWG outside of Institute of Medicine guidelines, and placental abruption, but it was also associated with a lower risk of GD. The findings suggest a complex association between prenatal cannabis use and maternal health and highlight the need for continued research to understand the mechanisms through which prenatal cannabis use is associated with the health of pregnant individuals. Prenatal cannabis use is a risk factor for adverse neonatal outcomes.^6,7,8^ As we continue to learn about the potential harms and benefits of prenatal cannabis use, clinicians must provide coordinated, nonstigmatizing care and education to support pregnant individuals in making informed decisions about cannabis use.^68^

## Notes

Supplement 1. eFigure. STROBE Diagram of Patient Selection eMethods 1. Screening Methods for Prenatal Substance Use eMethods 2. Lists of ICD-9/10 Diagnosis Codes and Other Diagnostic Criteria Used to Identify Maternal Health Outcomes During Pregnancy eTable 1. Socio-Demographic Characteristics of Individuals Included Versus Excluded for Missing Prenatal Cannabis Use Data eTable 2. Pregnancy Characteristics, Overall and by Frequency of Prenatal Cannabis Use eTable 3. Risk Ratios of Maternal Outcomes by Any Prenatal Cannabis Use with Adjustment in Steps eTable 4. Sensitivity Analyses

Supplement 2. Data sharing statement
