# Supplementary material for: Prenatal Cannabis Use and Maternal Pregnancy Outcomes
Source: JAMA Intern Med. 2024 Jul 22;184(9):1083–93. doi: 10.1001/jamainternmed.2024.3270 (PMC11264060; doi:10.1001/jamainternmed.2024.3270)
Supplement: Supplement 1. — eFigure. STROBE Diagram of Patient Selection eMethods 1. Screening Methods for Prenatal Substance Use eMethods 2. Lists of ICD-9/10 Diagnosis Codes and Other Diagnostic Criteria Used to Identify Maternal Health Outcomes During Pregnancy eTable 1. Socio-Demographic Characteristics of Individuals Included Versus Excluded for Missing Prenatal Cannabis Use Data eTable 2. Pregnancy Characteristics, Overall and by Frequency of Prenatal Cannabis Use eTable 3. Risk Ratios of Maternal Outcomes by Any Prenatal Cannabis Use with Adjustment in Steps eTable 4. Sensitivity Analyses [file jamainternmed-e243270-s001.pdf]

Supplemental Online Content

Young-Wolff KC, Adams SR, Alexeeff SE, et al. Prenatal cannabis use and maternal pregnancy outcomes. *JAMA Intern Med*. Published online July 22, 2024. doi:10.1001/jamainternmed.2024.3270

eFigure. STROBE Diagram of Patient Selection
eMethods 1. Screening Methods for Prenatal Substance Use
eMethods 2. Lists of *ICD-9/10* Diagnosis Codes and Other Diagnostic Criteria Used to Identify Maternal Health Outcomes During Pregnancy
eTable 1. Socio-Demographic Characteristics of Individuals Included Versus Excluded for Missing Prenatal Cannabis Use Data
eTable 2. Pregnancy Characteristics, Overall and by Frequency of Prenatal Cannabis Use
eTable 3. Risk Ratios of Maternal Outcomes by Any Prenatal Cannabis Use with Adjustment in Steps
eTable 4. Sensitivity Analyses

This supplemental material has been provided by the authors to give readers additional information about their work.

© 2024 Young-Wolff KC et al. *JAMA Internal Medicine.*

eFigure. STROBE Diagram of Patient Selection

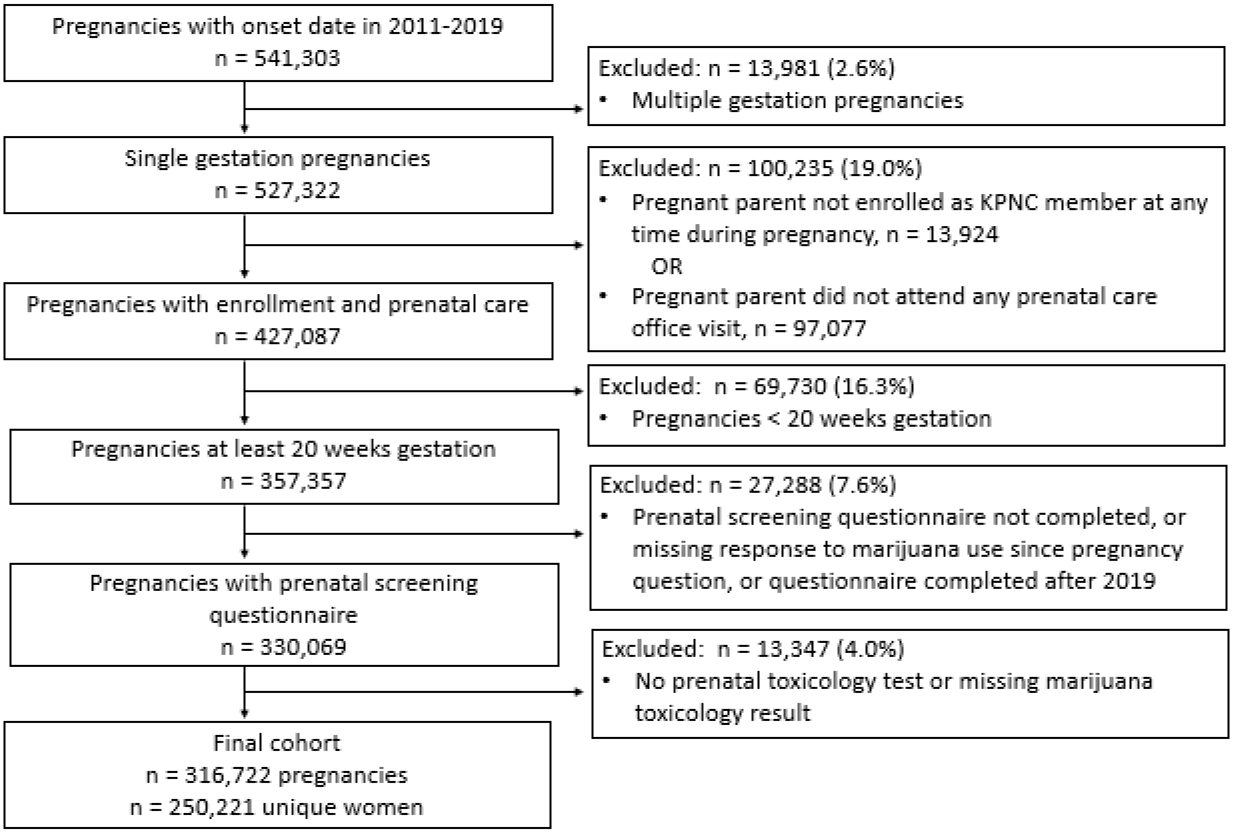

Abbreviation: KPNC, Kaiser Permanente Northern California.

© 2024 Young-Wolff KC et al. *JAMA Internal Medicine.*

eMethods 1. Screening Methods for Prenatal Substance Use

Prenatal Substance Use	Definition
Prenatal cannabis use	• Any use based on any self-reported use of cannabis during pregnancy at entrance to prenatal care and/or a positive toxicology test for tetrahydrocannabinol (THC)<br>• Any use based on a positive toxicology test (sensitivity analysis)<br>• Any use based on self-report (sensitivity analysis)<br>• Cannabis use frequency based on self-report (daily, weekly, monthly or less, never, or frequency unknown [i.e., reported no use but had a positive toxicology test])
Prenatal alcohol use	• Self-report for prenatal alcohol use at entrance to prenatal care<br>• Positive toxicology test for ethanol at entrance to prenatal care
Prenatal nicotine use	• Self-report of prenatal nicotine use (smoking cigarettes, vaping nicotine, or nicotine replacement therapy) at entrance to prenatal care<br>• Self-report of tobacco smoking at an office visit during pregnancy up to first prenatal visit date<br>• Nicotine replacement therapy prescription filled during pregnancy or before pregnancy with supply lasting past pregnancy onset date
Prenatal opioid use	• Self-report of prenatal heroin, methadone, or buprenorphine use at entrance to prenatal care<br>• Positive toxicology test for 6-monoacetylmorphine (6-MAM, heroin), morphine, codeine, hydrocodone, norhydrocodone, hydromorphone, oxycodone, nor oxycodone, or oxymorphone at entrance to prenatal care<br>• Opioid prescription filled during pregnancy or before pregnancy with supply lasting past pregnancy onset date
Prenatal stimulant use	• Self-report of prenatal cocaine, crack, or methamphetamine use at entrance to prenatal care<br>• Positive toxicology test for amphetamine, methamphetamine, MDMA (3,4-methylenedioxymethamphetamine), MDA (3,4-methylenedioxyamphetamine), or cocaine (benzoylecgonine) at entrance to prenatal care<br>• Amphetamine prescription filled during pregnancy or before pregnancy with supply lasting past pregnancy onset date
Prenatal anxiety or sleep medication use	• Self-report of prenatal sleep or anxiety medication use at entrance to prenatal care<br>• Positive toxicology test for benzodiazepines or barbiturates at entrance to prenatal care<br>• Anxiolytics or sedative prescription filled during pregnancy or before pregnancy with supply lasting past pregnancy onset date

Note: We used results from the first urine toxicology test during pregnancy ordered in the obstetrics and gynecology (OB-GYN) department typically at first prenatal visit (~8-10 weeks gestation). If no urine test

© 2024 Young-Wolff KC et al. *JAMA Internal Medicine.*

was ordered by the OB-GYN department, results from the toxicology test closest to 8 weeks gestation were used. The self-reported prenatal substance use screening and toxicology screenings are part of a standard initial panel of prenatal lab tests that are ordered for all patients. The standard panel of lab work is authorized by the clinician and entered by the medical assistant. The toxicology testing is opportunistic in that we are leveraging standard urine collection for toxicology tests, which is different from collecting a separate urine sample specifically for the sole purpose of toxicology. The urine sample collected for this standard prenatal lab testing includes other tests (e.g., urinalysis and culture) in addition to the toxicology testing. There is no decision making done by a provider to choose which patient gets a toxicology test and therefore no provider bias.

Information on Prenatal Urine Toxicology Tests by Substance:

THC/Cannabis: Cannabis screening tests were performed on a Beckman Coulter AU680 chemistry analyzer using the Emit II Plus Cannabinoid Assay from Siemens with a cutoff of 45ng/mL. Confirmatory testing for the presence of the cannabis metabolite, 11-nor-9-carboxy-delta 9- THC, was performed by liquid chromatography-tandem mass spectrometry for all positive immunoassay results. The confirmation test methodology was LC-MS/MS on a triple quadrupole system with a cutoff for positivity of 15ng/mL.

Alcohol: Alcohol screening tests were performed on a Beckman Coulter AU680 chemistry analyzer using the Emit II Plus Ethyl Alcohol Assay from Siemens with a cutoff of 10 mg/dL. Confirmatory testing for the presence of ethanol was performed by gas chromatography-mass spectrometry for all positive immunoassay results, also with a positive cutoff of 10 mg/dL.

Opioids: Opiate screening tests were performed on a Beckman Coulter AU680 chemistry analyzer using the Emit II Plus Opiate 300 Assay from Siemens with a cutoff of 300 ng/mL. Confirmatory testing for the presence of opiate species was performed by liquid chromatography- tandem mass spectrometry for all positive immunoassay results. Opiate species detected by the confirmation assay include morphine, 6-monoacetylmorphine (6-MAM), codeine, hydrocodone, norhydrocodone, hydromorphone, oxycodone, noroxycodone, and oxymorphone. All species have a positive cutoff of 50 ng/mL except for 6-MAM which has a positive cutoff of 10 ng/mL.

Amphetamine/Methamphetamine: Amphetamine screening tests were performed on a Beckman Coulter AU680 chemistry analyzer using the DRI Amphetamines Assay with a cutoff of 500 ng/mL. Confirmatory testing for the presence of amphetamine species was performed by liquid chromatography-tandem mass spectrometry for all positive immunoassay results. Amphetamine species detected by the confirmation assay include amphetamine, methamphetamine, MDMA, and MDA. All species have a positive cutoff of 250 ng/mL.

Cocaine: Cocaine screening tests were performed on a Beckman Coulter AU680 chemistry analyzer using the Emit II Plus Cocaine Metabolite Assay which detects the cocaine metabolite benzoylecgonine with a positive cutoff of 150 ng/mL. Confirmatory testing for the presence of benzoylecgonine was performed by liquid chromatography- tandem mass spectrometry for all positive immunoassay results. The positive cutoff for the confirmation assay was 100 ng/mL.

© 2024 Young-Wolff KC et al. *JAMA Internal Medicine.*

Barbiturates: Barbiturate screening tests were performed on a Beckman Coulter AU680 chemistry analyzer using the Emit II Plus Barbiturate Assay from Siemens with a cutoff of 180 ng/mL. Confirmatory testing for the presence of barbiturate species was performed by liquid chromatography-tandem mass spectrometry for all positive immunoassay results. Barbiturate species detected by the confirmation assay include butalbital and phenobarbital. All species have a positive cutoff of 100 ng/mL.

Benzodiazepines: Benzodiazepine screening tests were performed on a Beckman Coulter AU680 chemistry analyzer using the CEDIA Benzodiazepine Assay from Thermo Fisher Scientific with a cutoff of 200 ng/mL. This assay incorporates beta-glucuronidase treatment to detect total benzodiazepine species for those that have glucuronidated metabolites. Confirmatory testing for the presence of benzodiazepine species was performed by liquid chromatography-tandem mass spectrometry for all positive immunoassay results. Benzodiazepine species detected by the confirmation assay include alprazolam/alpha-hydroxyalprazolam, clonazepam/7-aminoclonazepam, flunitrazepam/7-aminoflunitrazepam, lorazepam, nordiazepam, oxazepam, temazepam, alpha-hydroxytriazolam, and zolpidem. All species have a positive cutoff of 25 ng/mL except for zolpidem which has a positive cutoff of 2.5 ng/mL.

© 2024 Young-Wolff KC et al. *JAMA Internal Medicine.*

eMethods 2. List of ICD-9/10 Diagnosis Codes and Other Diagnostic Criteria Used to Identify Maternal Health Outcomes During Pregnancy

Outcome	Definition
**Gestational hypertension**	Any of the following criteria during pregnancy:<br>1) ≥1 inpatient hospitalization with a diagnosis of hypertension;<br>2) ≥2 outpatient visits with a diagnosis of hypertension;<br>3) ≥1 outpatient visit with a diagnosis of hypertension and anti-hypertensives medication dispensing; or<br>4) 2 blood pressure measurements above 140/90 mm Hg on 2 occasions at least 4 hours apart<br>ICD-9: 642.3*, 642.9*<br>ICD-10: O13*, O16* (exclude O13.1, O16.1 - first trimester)
**Preeclampsia**	≥1 inpatient code during pregnancy<br>ICD-9: 642.40, 642.41, 642.43, 642.44, 642.50, 642.51, 642.52, 642.53, 642.54<br>ICD-10: O14, O14.0, O14.00, O14.02, O14.03, O14.04, O14.05, O14.1, O14.10, O14.12, O14.13, O14.14, O14.15, O14.2, O14.20, O14.22, O14.23, O14.24, O14.25, O14.9, O14.90, O14.92, O14.93, O14.94, O14.95
**Eclampsia**	≥1 inpatient code during pregnancy<br>ICD-9: 642.6, 642.60, 642.61, 642.62, 642.63, 642.64, 42.7<br>ICD-10: O15, O15.0, O15.00, O15.02, O15.03, O15.1, O15.2, O15.9
**Gestational diabetes (GDM)**	Individual is included in the KPNC Gestational Diabetes Registry as having diagnoses via Carpenter and Coustan (C&C criteria), lab, or doctor diagnoses from pregnancy onset to pregnancy outcome date or delivery inpatient discharge date for women who delivered in KPNC. Patients with pre-gestational diabetes and pregnancies that ended in therapeutic abortion were excluded.<br>• Carpenter and Coustan (C&C criteria) using the 3-hour glucose challenge test, i.e., if 2 or more from the 4 tests are above the threshold:<br>o Fasting >= 95 mg/dl;<br>o 1-hour >=180 mg/dl;<br>o 2-hour >= 155 mg/dl;<br>o 3-hour >=140 mg/dl;<br>• Lab: GDM if 1-h GCT >=180 mg/dl AND fasting glucose >=95 mg/dl<br>• Doctor diagnosis: Diagnosis of GDM using diagnostic codes; ICD codes included as "doctor diagnosis":<br>ICD-9: 648.80, 648.81, 648.82, 648.83, 648.84<br>ICD-10: O24.4, O24.41, O24.410, O24.414, O24.415, O24.419, O24.42, O24.420, O24.424, O24.425, O24.429, O24.43, O24.430, O24.434, O24.435, O24.439, O99.81, O99.810, O99.814, O99.815
**Gestational weight gain**	The 2009 Institute of Medicine trimester-specific recommendations for pregnancy weight gain. Range of recommended weight gain in 2nd and 3rd trimesters by pre-pregnancy body mass index (BMI) category by week:<br>o Underweight BMI category: 0.44–0.58 kg;<br>o Normal weight BMI category: 0.35–0.50 kg;<br>o Overweight BMI category: 0.23–0.33 kg;<br>o Obese BMI category: 0.17–0.27 kg.

© 2024 Young-Wolff KC et al. *JAMA Internal Medicine.*

	Assumes a 0.5 – 2.0 kg weight gain in the first trimester.
**Placenta previa**	≥1 inpatient ICD code that occurred during a KPNC inpatient/hospitalization stay from pregnancy onset to pregnancy end<br>ICD-9: 641.0, 641.00, 641.01, 641.03, 641.1, 641.10, 641.11, 641.13, 762.0<br>ICD-10: O44, O44.0, O44.00, O44.01, O44.02, O44.03, O44.1, O44.10, O44.11, O44.12, O44.13, O44.2, O44.20, O44.21, O44.22, O44.23, O44.3, O44.30, O44.31, O44.32, O44.33, O44.4, O44.40, O44.41, O44.42, O44.43, O44.5, O44.50, O44.51, O44.52, O44.53, P02.0
**Placental abruption**	≥1 inpatient or outpatient ICD code that occurred from pregnancy onset to pregnancy end<br>ICD-9: 641.2, 641.20, 641.21, 641.23<br>ICD-10: O45, O45.0, O45.00, O45.001, O45.002, O45.003, O45.009, O45.01, O45.011, O45.012, O45.013, O45.019, O45.02, O45.021, O45.022, O45.023, O45.029, O45.8, O45.8X, O45.8X1, O45.8X2, O45.8X3, O45.8X9, O45.9, O45.90, O45.91, O45.92, O45.93, O45.09, O45.091, O45.092, O45.093, O45.099
**Placental accreta**	Only identifiable for women who delivered at KPNC.<br>≥1 inpatient code<br>ICD-9: 667.0<br>ICD-10: O43.2, O43.21, O43.211, O43.212, O43.213, O43.219, O43.22, O43.221, O43.222, O43.223, O43.229, O43.23, O43.231, O43.232, O43.233, O43.239
**Severe maternal morbidity**	Centers for Disease Control and Prevention: https://www.cdc.gov/reproductivehealth/maternalinfanthealth/smm/severe-morbidity-ICD.htm.

Abbreviations:
ICD-9/10, International Classification of Diseases, Ninth Revision, Clinical Modification and International Statistical Classification of Diseases, Tenth Revision, Clinical Modification.
KPNC, Kaiser Permanente Northern California.

References:
Carpenter, MW, Coustan, DR. Criteria for screening tests for gestational diabetes. Am J Obstet Gynecol. 1982;144(7):768-773. doi:10.1016/0002-9378(82)90349-0
Centers for Disease Control and Prevention. How does CDC identify severe maternal morbidity? Accessed March 8, 2024, https://www.cdc.gov/reproductivehealth/maternalinfanthealth/smm/severe-morbidity-ICD.htm
Institute of Medicine and Natural Research Council. Weight Gain During Pregnancy: Reexamining the Guidelines. National Academies Press; 2009. https://doi.org/10.17226/12584

© 2024 Young-Wolff KC et al. *JAMA Internal Medicine.*

eTable 1. Socio-Demographic Characteristics of Pregnancies Included in Study Cohort Versus Excluded for Missing Prenatal Cannabis Use Data

	N (%)		
**Characteristics**	**Included in cohort** N = 316,722 (88.6%)	**Excluded from cohort** N = 40,635 (11.4%)	**Standardized difference**[a]
**Age**			0.05
<18	2,994 (0.9)	406 (1.0)	
18-24	46,021 (14.5)	6,218 (15.3)	
25-30	87,882 (27.8)	10,874 (26.8)	
31-35	113,020 (35.7)	14,030 (34.5)	
36+	66,805 (21.1)	9,107 (22.4)	
**Race and ethnicity**			0.09
Hispanic	83,145 (26.3)	10,905 (26.8)	
Non-Hispanic			
Asian/Pacific Islander	84,039 (26.5)	9,975 (24.6)	
Black	20,053 (6.3)	3,183 (7.8)	
White	118,333 (37.4)	14,832 (36.5)	
American Indian/Alaska Native/multi-racial/unknown	11,152 (3.5)	1,740 (4.3)	
**Neighborhood deprivation index**			0.33
Q1 - Least deprived	74,902 (23.7)	8,887 (21.9)	
Q2	74,934 (23.7)	8,724 (21.5)	
Q3	74,907 (23.7)	8,674 (21.4)	
Q4 - Most deprived	74,916 (23.7)	9,295 (22.9)	
Unknown	17,063 (5.4)	5,055 (12.4)	
**Insured by Medicaid**	28,803 (9.1)	4,244 (10.4)	-0.05

[a] Standardized difference = difference in proportions divided by standard error; imbalance defined as absolute value greater than 0.20 (small effect size).

© 2024 Young-Wolff KC et al. *JAMA Internal Medicine.*

eTable 2. Pregnancy Characteristics, Overall and by Frequency of Prenatal Cannabis Use

Pregnancy characteristics	No. (%)					
		Frequency of Prenatal Cannabis Use				
	Overall N=316,722	Daily N=1,930	Weekly N=2,345	Monthly or less N=4,892	Never N=296,669	Unknown frequency, positive toxicology N=10,886
Age at pregnancy onset						
<18	2,994 (0.9)	54 (2.8)	77 (3.3)	201 (4.1)	2,377 (0.8)	285 (2.6)
18-24	46,021 (14.5)	784 (40.6)	921 (39.3)	1,735 (35.5)	38,480 (13.0)	4,101 (37.7)
25-30	87,882 (27.7)	556 (28.8)	619 (26.4)	1,305 (26.7)	82,413 (27.8)	2,989 (27.5)
31-35	113,020 (35.7)	354 (18.3)	487 (20.8)	1,111 (22.7)	108,770 (36.7)	2,298 (21.1)
36+	66,805 (21.1)	182 (9.4)	241 (10.3)	540 (11.0)	64,629 (21.8)	1,213 (11.1)
Race and ethnicity						
Hispanic	83,145 (26.3)	450 (23.3)	534 (22.8)	1,354 (27.7)	77,562 (26.1)	3,245 (29.8)
Non-Hispanic						
Asian/Pacific Islander	84,039 (26.5)	80 (4.1)	142 (6.1)	423 (8.6)	82,848 (27.9)	546 (5.0)
Black	20,053 (6.3)	539 (27.9)	533 (22.7)	968 (19.8)	15,573 (5.2)	2,440 (22.4)
White	118,333 (37.4)	751 (38.9)	985 (42.0)	1,890 (38.6)	110,583 (37.3)	4,124 (37.9)
American Indian/Alaska Native/ multi-racial/unknown	11,152 (3.5)	110 (5.7)	151 (6.4)	257 (5.3)	10,103 (3.4)	531 (4.9)
Parity						
0	129,585 (40.9)	991 (51.3)	1,236 (52.7)	2,708 (55.4)	119,679 (40.3)	4,971 (45.7)
1	110,434 (34.9)	455 (23.6)	576 (24.6)	1,096 (22.4)	104,963 (35.4)	3,344 (30.7)
2+	65,908 (20.8)	338 (17.5)	353 (15.1)	764 (15.6)	62,500 (21.1)	1,953 (17.9)
Unknown	10,795 (3.4)	146 (7.6)	180 (7.7)	324 (6.6)	9,527 (3.2)	618 (5.7)
Prenatal care initiation						
Adequate plus (month 1-2)	198,083 (62.5)	1,134 (58.8)	1,258 (53.6)	2,607 (53.3)	186,151 (62.7)	6,933 (63.7)
Adequate (month 3-5)	99,481 (31.4)	641 (33.2)	834 (35.6)	1,743 (35.6)	92,862 (31.3)	3,401 (31.2)
Intermediate (month 5-6)	11,297 (3.6)	94 (4.9)	153 (6.5)	339 (6.9)	10,335 (3.5)	376 (3.5)
Inadequate (month 7+)	7,861 (2.5)	61 (3.2)	100 (4.3)	203 (4.1)	7,321 (2.5)	176 (1.6)
Insured by Medicaid	28,803 (9.1)	578 (29.9)	588 (25.1)	1,129 (23.1)	23,754 (8.0)	2,754 (25.3)
Neighborhood deprivation index						
Q1 - Least deprived	74,902 (23.6)	192 (9.9)	362 (15.4)	828 (16.9)	72,261 (24.4)	1,259 (11.6)
Q2	74,934 (23.7)	354 (18.3)	435 (18.6)	1,026 (21.0)	70,947 (23.9)	2,172 (20.0)
Q3	74,907 (23.7)	526 (27.3)	600 (25.6)	1,251 (25.6)	69,592 (23.5)	2,938 (27.0)
Q4 - Most deprived	74,916 (23.7)	803 (41.6)	855 (36.5)	1,613 (33.0)	67,406 (22.7)	4,239 (38.9)
Unknown	17,063 (5.4)	55 (2.8)	93 (4.0)	174 (3.6)	16,463 (5.5)	278 (2.6)
Non-cannabis substance use in pregnancy						
Alcohol	29,565 (9.3)	672 (34.8)	778 (33.2)	2,053 (42.0)	25,578 (8.6)	484 (4.4)

© 2024 Young-Wolff KC et al. *JAMA Internal Medicine.*

Nicotine	14,281 (4.5)	291 (15.1)	301 (12.8)	565 (11.5)	19,493 (6.6)	1,404 (12.9)
Opioids	22,054 (7.0)	121 (6.3)	119 (5.1)	208 (4.3)	1,114 (0.4)	150 (1.4)
Stimulants	1,712 (0.5)	773 (40.1)	806 (34.4)	1,197 (24.5)	9,782 (3.3)	1,723 (15.8)
Anxiety or sleep medications	9,163 (2.9)	209 (10.8)	213 (9.1)	420 (8.6)	7,793 (2.6)	528 (4.9)
Pre-pregnancy body mass index categories						
Underweight (<18.5 kg/m^2)	7,164 (2.3)	61 (3.2)	64 (2.7)	133 (2.7)	6,627 (2.2)	279 (2.6)
Normal (18.5-24.9 kg/m^2)	137,375 (43.4)	698 (36.2)	906 (38.6)	1,872 (38.3)	130,430 (44.0)	3,469 (31.9)
Overweight (25-29.9 kg/m^2)	83,426 (26.3)	495 (25.6)	595 (25.4)	1,278 (26.1)	78,151 (26.3)	2,907 (26.7)
Obese (30+ kg/m^2)	73,367 (23.2)	594 (30.8)	649 (27.7)	1,333 (27.2)	66,934 (22.6)	3,857 (35.4)
Unknown	15,390 (4.9)	82 (4.2)	131 (5.6)	276 (5.6)	14,527 (4.9)	374 (3.4)
Diagnoses and medications						
Period: two years before pregnancy						
Diabetes mellitus	4,026 (1.3)	27 (1.4)	20 (0.9)	45 (0.9)	3,804 (1.3)	130 (1.2)
Period: year before pregnancy through 1st prenatal visit						
Mood/anxiety disorder	34,984 (11.0)	542 (28.1)	564 (24.1)	1,090 (22.3)	30,470 (10.3)	2,318 (21.3)
Other psychiatric disorder	7,498 (2.4)	153 (7.9)	178 (7.6)	335 (6.8)	6,229 (2.1)	603 (5.5)
Substance use disorder diagnosis (other than cannabis)	11,220 (3.5)	504 (26.1)	502 (21.4)	740 (15.1)	7,969 (2.7)	1,505 (13.8)
Period: pregnancy onset through 1st prenatal visit						
Nausea and vomiting	35,639 (11.3)	499 (25.9)	510 (21.7)	876 (17.9)	30,997 (10.4)	2,757 (25.3)
Antidepressant medication use	6,441 (2.0)	125 (6.5)	122 (5.2)	192 (3.9)	5,576 (1.9)	426 (3.9)
Frequency of cannabis use						
Daily	1,930 (0.6)	1,930 (100.0)	0 (0.0)	0 (0.0)	0 (0.0)	0 (0.0)
Weekly	2,345 (0.7)	0 (0.0)	2,345 (100.0)	0 (0.0)	0 (0.0)	0 (0.0)
Monthly or less	4,892 (1.5)	0 (0.0)	0 (0.0)	4,892 (100.0)	0 (0.0)	0 (0.0)
Never	296,669 (93.7)	0 (0.0)	0 (0.0)	0 (0.0)	296,669 (100.0)	0 (0.0)
Unknown frequency, positive toxicology	10,886 (3.4)	0 (0.0)	0 (0.0)	0 (0.0)	0 (0.0)	10,886 (100.0)
Fetal outcome						
Live birth	314,480 (99.3)	1,917 (99.3)	2,329 (99.3)	4,835 (98.8)	294,600 (99.3)	10,799 (99.2)
Stillbirth	1,510 (0.5)	9 (0.5)	13 (0.6)	34 (0.7)	1,396 (0.5)	58 (0.5)
Therapeutic abortion	732 (0.2)	4 (0.2)	3 (0.1)	23 (0.5)	673 (0.2)	29 (0.3)

© 2024 Young-Wolff KC et al. *JAMA Internal Medicine.*

eTable 3: Risk Ratios of Maternal Outcomes by Any Prenatal Cannabis Use with Adjustment in Steps (N = 316,722)

	Risk ratios (95% CI) of maternal outcomes for any prenatal cannabis use (vs. none)[a]				
**Maternal outcomes**	**Model 1**	**Model 2**	**Model 3**	**Model 4**	**Model 5**
**Metabolic outcomes**					
**Hypertensive disorders[b]**					
Gestational hypertension	**1.42 (1.38-1.46)**	**1.30 (1.26-1.34)**	**1.20 (1.17-1.24)**	**1.17 (1.13-1.20)**	**1.17 (1.13-1.21)**
Preeclampsia	**1.39 (1.31-1.47)**	**1.25 (1.18-1.33)**	**1.10 (1.04-1.17)**	**1.08 (1.02-1.15)**	**1.08 (1.01-1.15)**
Eclampsia	**1.46 (1.03-2.08)**	1.23 (0.85-1.77)	1.13 (0.79-1.62)	1.18 (0.80-1.73)	1.17 (0.80-1.71)
**Gestational diabetes[c]**	**0.64 (0.61-0.68)**	**0.94 (0.89-0.99)**	**0.90 (0.86-0.95)**	**0.89 (0.85-0.94)**	**0.89 (0.85-0.94)**
**Gestational weight gain[d]**					
Within guidelines	NA	NA	NA	NA	NA
Below guidelines	**1.18 (1.15-1.21)**	**1.10 (1.06-1.13)**	**1.07 (1.04-1.10)**	**1.06 (1.03-1.09)**	**1.05 (1.01-1.08)**
Above guidelines	**1.15 (1.15-1.16)**	**1.11 (1.10-1.12)**	**1.11 (1.10-1.12)**	**1.09 (1.08-1.10)**	**1.09 (1.08-1.10)**
**Placental outcomes**					
Placenta previa	**0.75 (0.64-0.88)**	1.04 (0.89-1.21)	1.04 (0.89-1.22)	1.03 (0.87-1.21)	1.02 (0.87-1.20)
Placental abruption	1.12 (0.99-1.26)	**1.21 (1.07-1.37)**	**1.22 (1.08-1.38)**	**1.21 (1.06-1.38)**	**1.19 (1.05-1.36)**
Placenta accreta[e]	1.20 (0.86-1.67)	1.28 (0.91-1.82)	1.36 (0.96-1.93)	1.35 (0.93-1.96)	1.34 (0.92-1.95)
**Severe maternal morbidity**	**1.10 (1.02-1.19)**	1.06 (0.98-1.14)	1.03 (0.95-1.11)	0.99 (0.91-1.07)	0.97 (0.89-1.05)

Note: Bold values denote statistical significance at the *P* <.05 level.

[a] Model 1: Modified Poisson model with robust standard errors (no covariates)
Model 2: Adjusted for maternal sociodemographic characteristics (age category, race and ethnicity, neighborhood deprivation index)
Model 3: Additionally adjusted for parity, birth year, prenatal care initiation, pre-pregnancy body mass index category.
Model 4: Additionally adjusted for non-cannabis prenatal substance use (alcohol, nicotine, opioids, stimulants, and anxiety/sleep medication)
Model 5: Additionally adjusted for maternal medical and mental health comorbidities (pre-gestational diabetes mellitus, nausea/vomiting during pregnancy, mood/anxiety disorders, other psychiatric disorders, substance use disorders [other than cannabis], antidepressant use)

[b] Pregnancies to individuals with chronic hypertension were excluded (n = 14,187).

[c] Pregnancies to individuals with pre-gestational diabetes mellitus were excluded. In addition, gestational diabetes could not be ascertained for pregnancies ending in therapeutic abortion. Total excluded = 4,737. In Model 5, pre-gestational diabetes mellitus was not included as a covariate.

[d] Categories determined by the 2009 Institute of Medicine guidelines. Pregnancies with missing weight values were excluded (n = 15,863). Below guidelines models were fit among pregnancies below or within guidelines; above guidelines models were fit among pregnancies above or within guidelines.

[e] Placenta accreta could only be ascertained for pregnancies that were delivered in a Kaiser Permanente Northern California facility and ended in live birth or stillbirth (alive at admission); 10,979 were excluded.

© 2024 Young-Wolff KC et al. *JAMA Internal Medicine.*

eTable 4: Sensitivity Analyses

Maternal outcomes	Risk ratios (95% CI) of maternal outcomes of any prenatal cannabis use (vs. none)[a]				
	Model 1 (unadjusted)		Model 2 (adjusted)		
	#1. Subset to patients without evidence of non-cannabis prenatal substance use (n=252,249)	#2. Subset to patients starting prenatal care in the first trimester (n=284,250)	#1. Subset to patients without evidence of non-cannabis prenatal substance use (n=252,249)	#2. Subset to patients starting prenatal care in the first trimester (n=284,250)	#3. Calendar year not included as covariate (n= 316,722)
**Metabolic outcomes**					
**Hypertensive disorders[b]**					
Gestational hypertension	**1.48 (1.42-1.56)**	**1.43 (1.39-1.48)**	**1.24 (1.18-1.30)**	**1.17 (1.13-1.21)**	**1.15 (1.11-1.19)**
Preeclampsia	**1.74 (1.59-1.90)**	**1.43 (1.34-1.52)**	**1.21 (1.10-1.33)**	**1.10 (1.03-1.18)**	**1.13 (1.06-1.20)**
Eclampsia	**1.88 (1.22-2.89)**	1.27 (0.84-1.90)	1.42 (0.91-2.22)	1.01 (0.65-1.55)	1.18 (0.81-1.73)
**Gestational diabetes[b]**	**0.58 (0.54-0.63)**	**0.66 (0.63-0.70)**	**0.83 (0.78-0.90)**	**0.90 (0.85-0.95)**	**0.87 (0.82-0.91)**
**Gestational weight gain[b]**					
Within guidelines	NA	NA	NA	NA	NA
Below guidelines	**1.20 (1.15-1.25)**	**1.17 (1.14-1.21)**	**1.05 (1.01-1.09)**	**1.04 (1.01-1.08)**	**1.05 (1.02-1.08)**
Above guidelines	**1.17 (1.16-1.18)**	**1.16 (1.15-1.17)**	**1.11 (1.10-1.12)**	**1.10 (1.09-1.11)**	**1.09 (1.08-1.10)**
**Placental outcomes**					
Placenta previa	**0.71 (0.57-0.88)**	**0.72 (0.61-0.85)**	1.01 (0.81-1.25)	0.98 (0.82-1.17)	1.03 (0.88-1.22)
Placental abruption	1.12 (0.95-1.32)	1.11 (0.97-1.26)	**1.25 (1.05-1.48)**	**1.18 (1.03-1.36)**	**1.19 (1.04-1.35)**
Placenta accreta[e]	0.97 (0.83-1.12)	1.07 (0.73-1.56)	1.05 (0.89-1.22)	NA	1.28 (0.88-1.86)
**Severe maternal morbidity**	**1.15 (1.03-1.28)**	1.08 (0.99-1.17)	1.03 (0.92-1.16)	0.94 (0.86-1.03)	0.97 (0.90-1.05)

Note: Bold values denote statistical significance at the *P* <.05 level.

[a] Model 1: Modified Poisson model with robust standard errors (no covariates). Model 2: Adjusted for maternal sociodemographic characteristics (age category, race and ethnicity, neighborhood deprivation index), parity, birth year, prenatal care initiation, pre-pregnancy body mass index category, non-cannabis prenatal substance use (alcohol, nicotine, opioids, stimulants, and anxiety/sleep medications), maternal medical and mental health comorbidities (pre-gestational diabetes mellitus, nausea/vomiting during pregnancy, mood/anxiety disorders, other psychiatric disorders, substance use disorders [other than cannabis], antidepressant use).

[b] Pregnancies to individuals with chronic hypertension were excluded.

[c] Pregnancies to individuals with pre-gestational diabetes mellitus were excluded. In addition, gestational diabetes could not be ascertained for pregnancies ending in therapeutic abortion. In Model 2, pre-gestational diabetes mellitus was not included as a covariate for the gestational diabetes outcome.

[d] Categories determined by the 2009 Institute of Medicine guidelines. Pregnancies with missing weight values were excluded. Below guidelines models were fit among pregnancies below or within guidelines; above guidelines models were fit among pregnancies above or within guidelines.

[e] Placenta accreta could only be ascertained for pregnancies that were delivered in a Kaiser Permanente Northern California facility and ended in live birth or stillbirth (alive at admission); The adjusted model subset to patients starting care in the first trimester did not converge.

© 2024 Young-Wolff KC et al. *JAMA Internal Medicine.*
