# Supplementary material for: Prenatal Cannabis Use and Maternal Pregnancy Outcomes
Source: JAMA Intern Med. 2024 Jul 22;184(9):1083–93. doi: 10.1001/jamainternmed.2024.3270 (PMC11264060; doi:10.1001/jamainternmed.2024.3270)
Supplement: Supplement 2. — Data sharing statement [file jamainternmed-e243270-s002.pdf]

Data Sharing Statement

Young-Wolff. Prenatal Cannabis Use and Maternal Pregnancy Outcomes. *JAMA Intern Med*. Published July 22, 2024. doi:10.1001/jamainternmed.2024.3270

Data
Data available: No
